# Th17 Cells Are More Protective Than Th1 Cells Against the Intracellular Parasite *Trypanosoma cruzi*

**DOI:** 10.1371/journal.ppat.1005902

**Published:** 2016-10-03

**Authors:** Catherine W. Cai, Jennifer R. Blase, Xiuli Zhang, Christopher S. Eickhoff, Daniel F. Hoft

**Affiliations:** 1 Department of Molecular Microbiology & Immunology, Saint Louis University School of Medicine, St. Louis, Missouri, USA; 2 Division of Infectious Diseases, Department of Internal Medicine, Saint Louis University School of Medicine, St. Louis, Missouri, USA; Ribeirão Preto School of Medicine - University of São Paulo - Ribeirão Preto, Brazil, BRAZIL

## Abstract

Th17 cells are a subset of CD4^+^ T cells known to play a central role in the pathogenesis of many autoimmune diseases, as well as in the defense against some extracellular bacteria and fungi. However, Th17 cells are not believed to have a significant function against intracellular infections. In contrast to this paradigm, we have discovered that Th17 cells provide robust protection against *Trypanosoma cruzi*, the intracellular protozoan parasite that causes Chagas disease. Th17 cells confer significantly stronger protection against *T*. *cruzi*-related mortality than even Th1 cells, traditionally thought to be the CD4^+^ T cell subset most important for immunity to *T*. *cruzi* and other intracellular microorganisms. Mechanistically, Th17 cells can directly protect infected cells through the IL-17A-dependent induction of NADPH oxidase, involved in the phagocyte respiratory burst response, and provide indirect help through IL-21-dependent activation of CD8^+^ T cells. The discovery of these novel Th17 cell-mediated direct protective and indirect helper effects important for intracellular immunity highlights the diversity of Th17 cell roles, and increases understanding of protective *T*. *cruzi* immunity, aiding the development of therapeutics and vaccines for Chagas disease.

## Author Summary

Chronic infection with the intracellular parasite *Trypanosoma cruzi* results in Chagas disease, an illness endemic in more than 20 countries that leads to life-threatening cardiac and gastrointestinal dysfunction. Although CD4^+^ Th1 cells are known to be protective against *T*. *cruzi* infection, less is known about the role of other CD4^+^ T cell subsets. We demonstrate that CD4^+^ Th17 cells are also highly protective against *T*. *cruzi* infection, even outperforming Th1 cells in protection from *T*. *cruzi*-related death, despite the standard conception that Th17 cells are only important in the defense against extracellular pathogens. The novel discovery that Th17 cells are significantly more protective than Th1 cells against *T*. *cruzi* infection has increased our understanding of the advantageous immune responses for this major human pathogen, and may guide future efforts toward vaccine development.

## Introduction

Chronic infection with the protozoan parasite *Trypanosoma cruzi* results in Chagas disease, a Neglected Tropical Disease currently affecting 8–11 million people worldwide and 300,000 people in the United States [1]. Humans usually acquire *T*. *cruzi* infection via reduviid insect vectors, but infections also sometimes occur through vertical transmission, the ingestion of contaminated food products, and the receipt of infected biological donations. The disease can cause significant cardiac and gastrointestinal morbidity, but drug therapies are typically non-curative and poorly tolerated [1]. The significant global burden of Chagas disease, coupled with the inefficacy of available treatments, indicates a pressing need to develop novel therapeutics, including preventative and/or therapeutic vaccines to induce protective *T*. *cruzi* immunity in at risk individuals. The development of effective vaccines requires a more detailed understanding of the protective host immune responses against *T*. *cruzi* infection.

Because *T*. *cruzi* promiscuously infects both cells expressing MHC class II and cells expressing only MHC class I, CD8^+^ T cells are critical for protection against infection of all host targets cells. However, CD4^+^ T cells also are needed for optimal protection [2]. CD4^+^ Th1 cells have been shown to provide both systemic and mucosal protection against *T*. *cruzi* infection, consistent with the well-established framework of Th1 cells being the CD4^+^ T cell subset most important against intracellular pathogens [3,4]. Less is known about the role of other CD4^+^ T cell subsets during *T*. *cruzi* infection.

More recently, studies have found a protective role for IL-17A, the major cytokine produced by CD4^+^ Th17 cells, raising questions about the possibility of a protective role for Th17 cells [5–7]. However, multiple subtypes of IL-17-producing cells exist, including αβ T cells, γδ T cells, innate lymphoid cells, and even B cells in *T*. *cruzi* infection [8,9]. In addition, Th17 cells have been shown to be involved in autoimmunity [10–12]. Thus, it remained unclear from these studies of global IL-17A deficiency whether Th17 cells specifically play a protective or pathologic role in *T*. *cruzi* immunity. Although Th17 cells are now known to protect against certain extracellular bacteria and fungi [13–16], they are not thought to have a significant function in intracellular immunity.

To investigate the specific effects of different CD4^+^ T cell subsets in *T*. *cruzi* infection, we generated T cell receptor transgenic (TS-CD4-Tg) mice with CD4^+^ T cell receptors that are specific for p7, an immunodominant epitope encoded by the *T*. *cruzi trans*-sialidase, a highly conserved virulence factor and potential vaccine target (S1 and S2 Figs) [17–20]. Using CD4^+^ T cells from these mice, we were able to generate parasite-specific Th1 and Th17 cells *in vitro*. These cells were used for studies *in vivo* using adoptive transfer experiments, and *in vitro* using CD8^+^ T cell activation and macrophage infection assays. Through these experiments, we found that Th17 cells confer robust protection against *T*. *cruzi* infection, even surpassing that provided by Th1 cells, through both direct and indirect protective effects.

## Results

### Th17 cells provide stronger protection from *T*. *cruzi-*related mortality than Th1 cells

**Fig 1:**
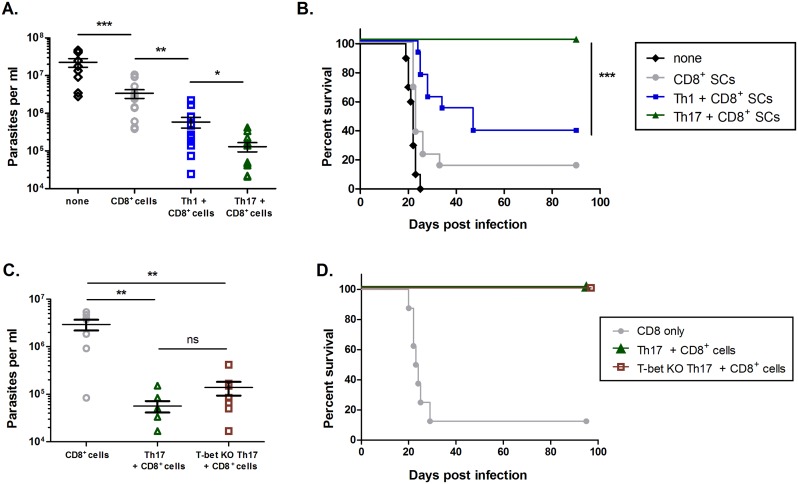
Th17 cells provide better protection *in vivo* than Th1 cells. Polyclonal CD8^+^ T cells were transferred into RAG KO mice along with either Th1 or Th17 *T*. *cruzi*-specific T cells (>10/group). Mice were then systemically challenged with *T*. *cruzi*. (A) Parasitemia counts 19 days post-infection demonstrate that co-adoptive transfer of Th17 cells resulted in better control of infection than co-adoptive transfer of Th1 cells ***p<0.001, **p<0.01, *p<0.05 by two-tailed Student t test. (B) All groups receiving CD8^+^ T cells were significantly protected compared to no adoptive transfer. Co-adoptive transfer of Th17 cells led to 100% long-term survival that was significantly higher than survival in all other groups.***p<0.001 by Mantel-Cox log-rank test. (C) T-bet KO Th17 cells resulted in decreases in parasite load comparable to WT Th17 cells when transferred into RAG KO mice (>7/group) with CD8^+^ T cells. **p<0.01 by two-tailed Student t test. (D) T-bet KO Th17 cells resulted in equivalent long-term protection from mortality compared to WT Th17 cells.

To study the roles of Th1 cells versus Th17 cells *in vivo*, *in vitro*-differentiated TS-CD4-Tg Th1 or Th17 cells were adoptively transferred with naïve polyclonal CD8^+^ T cells into RAG KO mice, which were then challenged subcutaneously with *T*. *cruzi* (S3 Fig, Fig 1A and 1B). We confirmed the persistence of these transferred cells (S4 Fig). As expected, control RAG KO mice (no T cell transfer) did not survive the challenge, and nearly all mice receiving CD8^+^ T cells alone also succumbed to infection. Consistent with previous studies supporting a protective function for Th1 cells against *T*. *cruzi*, mice receiving co-transfer of Th1 cells and CD8^+^ T cells exhibited lower blood parasite burdens and improved long-term survival. However, surprisingly, mice receiving co-transfer of Th17 cells and CD8^+^ T cells experienced the strongest protection, as indicated by the lowest blood parasite burdens and highest survival rates (100% long term) of any group. This represented significantly improved protection even compared to mice receiving Th1 cells (p<.001 comparing Th17 and CD8^+^ T cells to Th1 and CD8^+^ T cells, Fig 1A and 1B).

Because our Th17 cell cultures also contained undifferentiated cells not expressing the classical Th17 markers RORγt or IL-17 (S3C Fig), we sought to confirm that the impressive protection was truly a Th17 cell-mediated effect, and not attributable to another cell type present in the Th17 cell cultures. To do so, we used cell purification kits to obtain >90% purity of IL-17^+^ cells from our Th17 cell cultures (S5A Fig). Co-transfer of these cells with polyclonal CD8^+^ T cells into RAG KO mice resulted in sustained Th17 cell responses and similar activation of the CD8^+^ T cells (S5B and S5C Fig). Even more importantly, the transfer of highly purified IL-17^+^ T cells resulted in protection from *T*. *cruzi* (S5D and S5E Fig), confirming that bona fide Th17 cells provide protection against this parasite. We also tested polyclonal *T*. *cruzi-*specific Th17 cells, which were generated by stimulating wild-type CD4^+^ T cells with dendritic cells pulsed with whole parasite lysate antigens under Th17-skewing conditions (S6A Fig). We found that polyclonal Th17 cells, which better model a Th17 cell response that could arise *in vivo*, also result in significantly lower parasitemia and 100% long-term survival when given with CD8^+^ T cells (S6B–S6D Fig).

Furthermore, Th17 cells have been reported to maintain plasticity and revert to Th1 phenotypes *in vivo* [21–23], raising another important consideration. Indeed, although Th17 cells persisted after transfer, as indicated by sustained production of IL-17, a small percentage of *in vitro-*biased Th17 cells expressed IFN-γ (S3C Fig), and some Th1 cells emerged from Th17 cells *in vivo* (S4A and S4C Fig). Therefore, we asked whether the IFN-γ expressed by a small subset of Th17 cells, or the trans-differentiation of Th17 cells into Th1 cells after transfer, was responsible for the protective effects. To answer this question, we generated T-bet KO TS-CD4-Tg mice lacking T-bet, the transcription factor that drives Th1 differentiation and IFN-γ production (T-bet KO TS-CD4-Tg). Using CD4^+^ T cells from these mice, we made *T*. *cruzi*-specific T-bet KO Th17 cells, which are unable to adopt the Th1 phenotype. We gave RAG KO mice T-bet KO TS-CD4-Tg Th17 cells and polyclonal CD8^+^ T cells, and infected them with *T*. *cruzi*. CD4^+^ T cells recovered from recipient mice 9 and 101 days after infection confirmed that the transferred T-bet KO Th17 cells were veritable Th17 cells, as they did not produce IFN-γ *in vivo*, but maintained high levels of IL-17A expression (S7A and S7B Fig). Mice receiving WT or T-bet KO Th17 cells were similarly protected, with significantly decreased parasite burdens compared to control and 100% long-term survival (Fig 1C and 1D). The persistence of Th17 cells and their protective effects could be detected more than three months after initial infection (S7C Fig), providing further evidence that a stable Th17 cell response to *T*. *cruzi* is possible, and that there is no requirement for Th1 cells or any other IFN-γ^+^ CD4^+^ T cells for robust *T*. *cruzi* immunity.

### IL-17A induces NADPH oxidase function to provide direct protection *in vitro*

**Fig 2:**
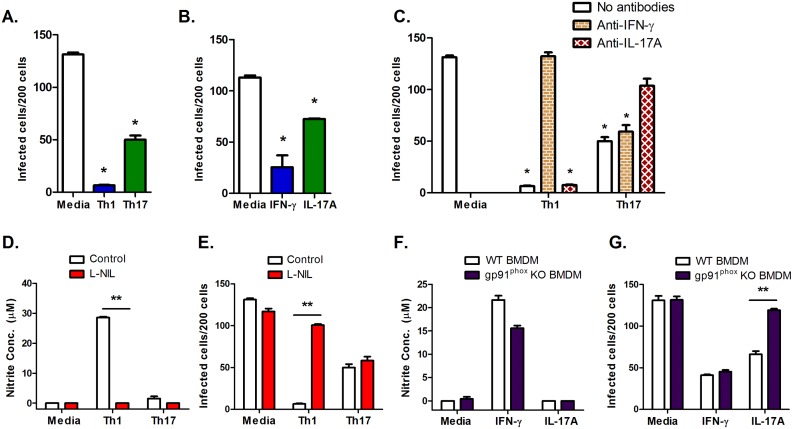
Th17 cells can inhibit *T*. *cruzi* intracellular growth in macrophages *in vitro* by inducing NADPH oxidase via IL-17A. PEMs were infected with trypomastigotes for 3 hours (MOI = 5). *T*. *cruzi*-specific TCR Tg Th1 or Th17 cells were then added to macrophages 1:25. Two days later, slides were Giemsa stained and intracellular parasites counted microscopically. Th1 and Th17 cells (A), as well as IFN-γ or IL-17A alone (B) were able to decrease the number of infected macrophages. *p<0.001 compared to medium alone by two-tailed Student t test. (C) Neutralizing antibodies directed against IFN- γ or IL-17A were able to abolish the direct protection provided by Th1 and Th17 cells, respectively. (D-E) The effects of the iNOS inhibitor L-NIL on Th1- and Th17-induced NO production and *T*. *cruzi* protection are shown. **p<0.001 by two-tailed Student t test. (F+G) BMDMs from wild type (WT) and gp91^phox^ KO mice were infected with *T*. *cruzi* for 3 hours (MOI = 10), then cytokines IFN-γ and IL-17A were added. Two days later, NO was measured (F) and intracellular parasites were enumerated (G). **p<0.001 by two-tailed Student t test. These results were reproduced in multiple experiments.

To investigate mechanisms of direct protection against *T*. *cruzi* infection by Th17 cells, we infected murine peritoneal exudate macrophages (PEM) with *T*. *cruzi* trypomastigotes *in vitro* and added Th1 or Th17 cells for two days. In the absence of T cells, high-level parasite infection occurred. The addition of either Th1 cells or purified IFN-γ alone significantly reduced numbers of cells that became infected after two days. Th1 cells are known to prime macrophage activation for the killing of intracellular microorganisms through the secretion of IFN-γ. Consistent with this, a neutralizing antibody directed against IFN-γ reversed the effects of Th1-mediated protection (Fig 2A–2C) [24]. The addition of Th17 cells to the cultures also reduced the parasite burden among infected macrophages, indicating that Th17 cells are able to directly protect cells against *T*. *cruzi* infection. This effect could be recapitulated by replacing Th17 cells with purified IL-17 cytokine, or abrogated by adding an IL-17A-neutralizing antibody, indicating that the protection is mediated by the IL-17 produced by Th17 cells. Although Th17-derived IL-17A is known to recruit neutrophils and enhance inflammation in extracellular infections, a direct intracellular protective effect has not been previously reported.

We confirmed previous studies showing that Th1 cells, through the production of IFN-γ, induce macrophage iNOS to produce nitric oxide [25], the mechanism underlying the killing of intracellular pathogens. However, NO was not induced by Th17 cells (Fig 2D), and although the addition of the iNOS inhibitor N6-(1-Iminoethyl)-L-lysine (L-NIL) reversed Th1-mediated protection, it had no effect on Th17-mediated protection (Fig 2E). These data indicate that the direct protection provided by Th17 cells operates independently of NO generation.

Similar to NO-mediated protection, ROS can also inhibit the growth of intracellular pathogens in neutrophils and macrophages [26]. IL-17A has been reported to induce production of ROS in endothelial cells, but it was unknown whether the cytokine could induce ROS in other cells [27]. We hypothesized that Th17 cells could also induce ROS production in macrophages, and that the induction of ROS was necessary for IL-17-mediated direct protection. To test our hypothesis, we infected bone marrow-derived macrophages (BMDMs) generated from WT and gp91^phox^ KO mice. The latter are genetically deficient in a critical subunit of the NADPH oxidase, resulting in a defective phagocyte respiratory burst response. Following infection of these cells, we treated them with IFN-γ or IL-17A for 48 hours. The gp91^phox^ deficiency did not affect the induction of NO (Fig 2F). In both infected WT and gp91^phox^ KO BMDM, IFN-γ treatment induced NO production, but IL-17A did not (Fig 2F). In WT BMDM, both IFN-γ and IL-17A protected against infection and intracellular growth of *T*. *cruzi*. Deficiency of the gp91^phox^ subunit of NADPH oxidase had no effect on IFN-γ-mediated protection, but reversed IL-17A-mediated protection, indicating that functional NADPH oxidase is required for this mechanism, and suggesting the involvement of ROS generated during the respiratory burst (Fig 2G).

### IL-17A does not mediate protection by Th17 cells *in vivo*

**Fig 3:**
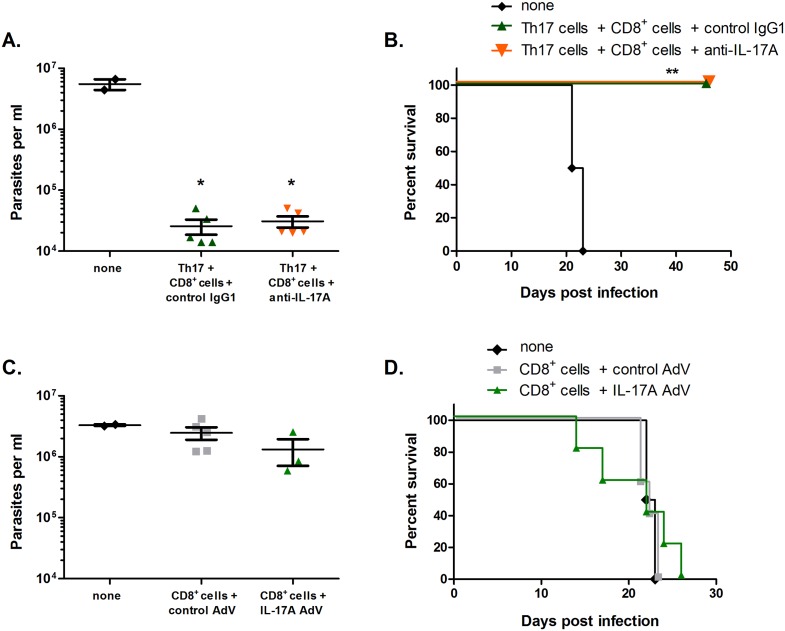
IL-17A alone is not responsible for the Th17-mediated enhanced protective effects. (A-B) *T*. *cruzi*-specific Th17 cells were co-transferred with CD8^+^ T cells into RAG KO mice (5/group) prior to *T*. *cruzi* challenge. Neutralizing anti-IL-17A or control IgG1 antibodies were injected intraperitoneally every 48 hours. IL-17A neutralization did not reduce protection as measured by both parasitemia (A) and survival (B). *p<0.001 by two-tailed Student t test, **p<0.01 by log-rank test compared with negative controls. (C-D) Polyclonal CD8^+^ T cells were transferred intravenously (i.v.) into RAG KO mice prior to *T*. *cruzi* infection. Either control AdV or IL-17 AdV was injected 1 day prior to infection and 7 days post-infection at 5x10^9^ PFU i.v. Protection was measured by parasitemia 18 days post-infection (C) and survival (D).

Given our findings concerning IL-17-mediated protection *in vitro*, we asked whether IL-17A was also responsible for the protection observed *in vivo*. We first neutralized IL-17A in RAG KO mice reconstituted with Th17 cells and polyclonal CD8^+^ T cells by injection of a monoclonal anti-IL-17A antibody previously shown to reverse IL-17A function *in vivo* [28,29]. Neutralizing IL-17A had no effect on parasitemia levels or survival percentages in these mice (Fig 3A and 3B). We also overexpressed IL-17A in RAG KO mice receiving polyclonal CD8^+^ T cells alone using a recombinant IL-17A-producing adenovirus (IL-17A AdV). Although mice injected with IL-17A AdV had markedly higher serum levels of IL-17A (28 ng/ml ± 16 in IL-17 AdV sera versus <0.02ng/ml in control AdV sera), overexpression of IL-17A alone was not enough to replace Th17 cells, and did not protect mice also receiving CD8^+^ T cells (Fig 3C and 3D). Overall, these results indicate that IL-17 is dispensable for the major *in vivo* protective effects induced by Th17 cells, despite being the major Th17 cytokine implicated in previous reports on Th17-based protection and pathology. Therefore, in *T*. *cruzi* infection, Th17 cells must function through an IL-17-independent mechanism to protect.

### Helper effects on CD8^*+*^ T cells execute Th17-mediated protection *in vivo*

**Fig 4:**
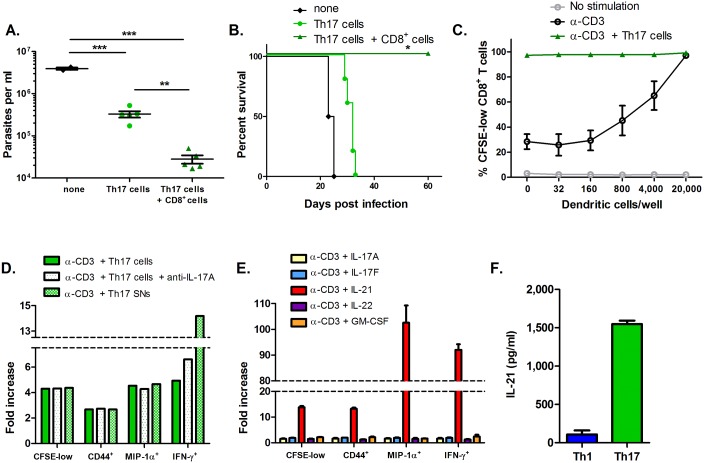
*T*. *cruzi*-specific Th17 cells provide T helper effects and directly activate CD8^+^ T cells through a soluble mediator. (A) Th17 cells required co-adoptive transfer with polyclonal CD8^+^ T cells to confer optimal immunity as measured by parasite burden. **p<0.01, ***p<0.001 by two-tailed Student t test. (B) Th17 cells transferred alone were not able to protect mice from *T*. *cruzi*-related death, indicating that Th17 cells protect through helper effects on CD8^+^ T cells. **p<0.01 by Mantel-Cox log-rank test. (C) CFSE-labeled, polyclonal CD8^+^ T cells were sub-optimally activated with plate-bound α-CD3 (1 μg/ml) and increasing numbers of dendritic cells. CD8^+^ T cells were cultured with or without Th17 cells for 5 days. Proliferation was measured by CFSE dilution. (D) CD8^+^ T cells were sub-optimally activated with α-CD3 as in C (without added dendritic cells), in the presence of Th17 cells, Th17 SNs or Th17 cells plus IL-17A neutralizing antibody. CD8^+^ T cell proliferation, CD44 expression and MIP-1α/IFN-γ production were measured 5 days later by Flow Cytometry and ICS and shown as fold increases compared with α-CD3 activation alone. (E) Purified Th17 cytokines were individually added to sub-optimally α-CD3 activated CD8^+^ T cells and markers of activation were analyzed 5 days later. (F) High levels of IL-21 could be detected by ELISA in Th17 cell SNs, but not in Th1 cell SNs. Representative results from multiple experiments are shown.

To discriminate between direct protective versus indirect T helper effects, we studied protection provided by Th17 cells transferred alone or with polyclonal CD8^+^ T cells. CD8^+^ T cells were needed for optimal Th17-mediated protection (Fig 4A and 4B), indicating Th17 helper effects for CD8^+^ T cells were most important *in vivo*. However, the mechanism of Th17 helper effects on CD8^+^ T cells could be due to Th17 cells directly instructing these cells, or Th17 cells indirectly affecting these cells via the activation of antigen-presenting cells. To distinguish between these potential mechanisms, naïve polyclonal CD8^+^ T cells were stimulated with suboptimal α-CD3 *in vitro*. Th17 cells and/or immature dendritic cells (DCs) were added to these cultures to provide potential co-stimulatory help. In the absence of Th17 cells, CD8^+^ T cells exhibited progressive degrees of proliferation with sub-optimal α-CD3 stimulation and increasing doses of DC (Fig 4C), as detected by dilution of the cell staining dye CFSE. However, nearly 100% of CD8^+^ T cells proliferated maximally when Th17 cells were introduced, even in the absence of DC. These data indicate that Th17 cells directly and strongly enhance the activation of naïve CD8^+^ T cells.

### Th17 cells exert T helper effects on CD8^+^ T cells via IL-21

In addition to increasing the proliferation of CD8^+^ T cells, Th17 cells also induced these cells to increase the expression of surface CD44 (a marker of activated T cells), the effector cytokine IFN-γ, and the chemokine MIP-1α, indicating an activated state of CD8^+^ T cells (Fig 4D). These helper effects on CD8^+^ T cells were also detectable using supernatants (SN) from activated Th17 cells, implicating soluble factors (Fig 4D). However, consistent with our previous *in vivo* results, neutralization of IL-17A did not prevent the Th17-mediated activation of CD8^+^ T cells *in vitro* (Fig 4D). By co-culturing sub-optimally stimulated CD8^+^ T cells with individual purified Th17 cytokines, we discovered that IL-21 alone recapitulated this potent help, inducing a state of CD8^+^ T cell activation similar to that seen with the addition of Th17 cells or Th17 cell SNs (Fig 4E). We confirmed that our Th17 cells produced significant amounts of IL-21, while the Th1 cells did not (Fig 4F, S3F Fig).

**Fig 5:**
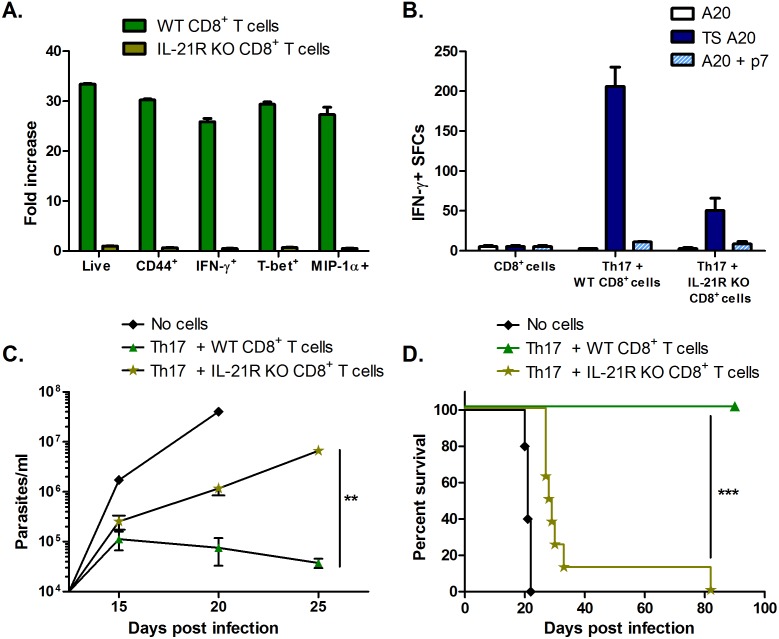
Th17 cell-derived IL-21 can help expand and activate protective CD8^+^ T cells. (A) WT or IL-21R KO CD8^+^ T cells were sub-optimally stimulated with plate-bound α-CD3 as in Fig 4C and 4E. ICS assays indicated the addition of Th17 cell SNs could help activate WT CD8^+^ T cells but not IL-21R KO CD8^+^ T cells. Shown are fold increases compared to WT or IL-21R KO CD8^+^ T cells cultured with sub-optimal α-CD3 alone, without Th17 cell SNs. (B) Total spleen cells (TSCs) recovered from infected RAG KO mice adoptively transferred with Th17 cells and IL-21R KO CD8^+^ T cells had diminished responses to TS stimulation compared to mice receiving Th17 cells and WT CD8^+^ T cells prior to challenge, as measured by IFN-γ ELISPOT. (C) Mice transferred with Th17 cells (n = 4) and IL-21R KO CD8^+^ T cells (n = 8) were unable to control infection, as indicated by increasing parasitemia over time. **p<0.01 by two-tailed Student t test. (D) Transfer of IL-21R KO CD8^+^ T cells failed to protect mice from *T*. *cruzi*-mortality. *** p<0.001 by Mantel-Cox log-rank test.

*In vitro* assays demonstrated that IL-21R KO CD8^+^ T cells are unable to be activated by Th17 cell SNs in the same manner as WT CD8^+^ T cells (Fig 5A), confirming the IL-21 signaling requirement for Th17-mediated protection. To test these results *in vivo*, we transferred Th17 cells into RAG KO mice with either WT or IL-21R KO CD8^+^ T cells, and challenged the mice with *T*. *cruzi*. Recovered spleen cells from mice receiving IL-21R KO CD8^+^ T cells with Th17 cells had impaired responses to *T*. *cruzi* TS antigen/peptide stimulation (Fig 5B). Furthermore, only mice receiving Th17 cells with WT CD8^+^ T cells were able to control infection. Although mice given WT CD8^+^ T cells reduced parasitemia after day 15, parasitemia levels steadily increased in mice receiving IL-21R KO CD8^+^ T cells (Fig 5C). As a result of this failed protection, all mice receiving IL-21R KO CD8^+^ T cells succumbed to infection while 100% of mice receiving WT CD8^+^ T cells with Th17 cells survived long-term (Fig 5D). These studies clearly demonstrate that Th17-mediated protection *in vivo* operates through the secretion of IL-21, and the resulting indirect T helper effects on CD8^+^ T cells.

### Th17 cells result in greater numbers of CD8^+^ T cells, higher levels of T-bet expression and stronger antigen-specific responses than Th1 cells

**Fig 6:**
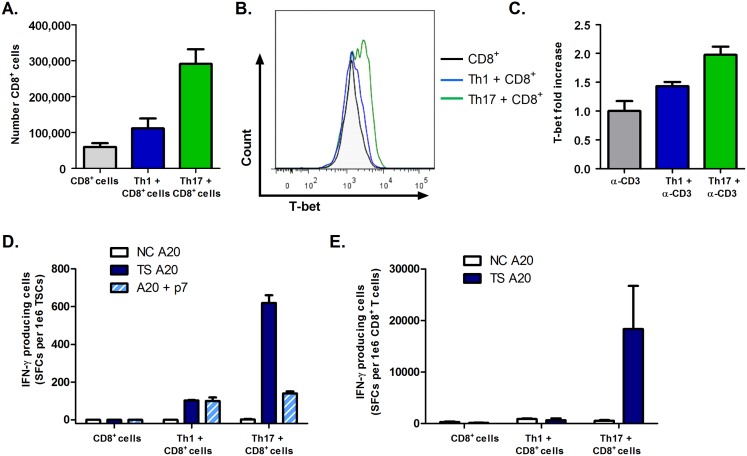
Enhanced protection by Th17 cells involves more potent qualitative and quantitative helper effects for the development of *T*. *cruzi*-specific CD8^+^ T cells. RAG KO mice were adoptively transferred with polyclonal CD8^+^ T cells and Th1 or Th17 cells and then infected systemically with *T*. *cruzi*. (A) Greater absolute numbers of CD8^+^ T cells were recovered from mice co-adoptively transferred with Th17 cells at 7 days post-infection. (B) The mean fluorescence intensity (MFI) of T-bet expression in these same CD8^+^ T cells was increased over mice receiving CD8^+^ T cells alone or CD8^+^ T cells with Th1 cells, as measured by ICS. (C) Co-culture of sub-optimally stimulated CD8^+^ T cells with activated Th17 cell SN *in vitro* also induced higher T-bet expression than co-culture with Th1 cell SN. (D-E) Antigen-specific total and CD8^+^ T cell responses were studied 7 days post-challenge by IFN-γ ELISPOT (D) and intracellular cytokine staining (E), respectively. Similar results were detected in multiple experiments.

Given the improved protection afforded by Th17 over Th1 helper cells, we next explored the mechanisms responsible for the reason Th17-induced CD8^+^ T cells can control infection better than Th1-induced CD8^+^ T cells. Recovered spleen cells from RAG KO mice reconstituted with CD8^+^ T cells and Th1 or Th17 cells prior to *T*. *cruzi* challenge demonstrated that mice receiving Th17 cells had greater expansion of CD8^+^ T cells than mice receiving Th1 cells (Fig 6A). In addition, CD8^+^ T cells primed by Th17 cells either *in vivo* (Fig 6B) or *in vitro* (Fig 6C) expressed higher levels of T-bet compared with Th1-primed cells, which has been associated with higher cytotoxic activity and IFN-γ production [30,31]. IFN-γ ELISPOT and ICS assays comparing spleen cells recovered from Th1- or Th17-transferred mice demonstrated that mice receiving Th17 cells also had >6-fold stronger total spleen cell responses to *T*. *cruzi* TS antigen, and >10,000-fold greater frequencies of antigen-specific CD8^+^ T cells than mice receiving Th1 cells (Fig 6D and 6E). Overall, these results indicate that both quantitative and qualitative differences distinguish Th1- and Th17-induced CD8^+^ T cells.

### Th17 cells also provide robust helper effects to CD8^+^ T cells and B cells in immunocompetent mice

**Fig 7:**
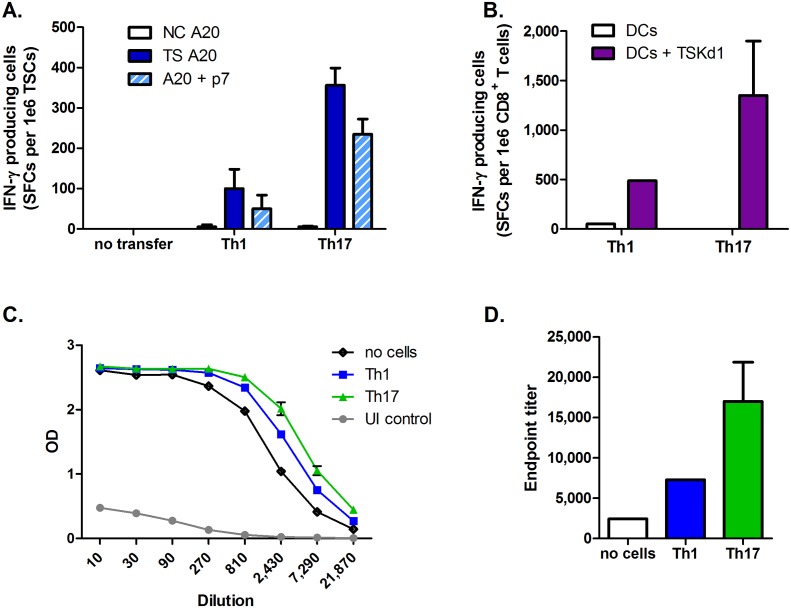
Th17 cells also provide robust helper effects to CD8^+^ T cells and B cells in immunocompetent mice. Total spleen cells (A) and purified CD8^+^ T cells (B) recovered from WT BALB/c mice receiving TS-CD4-Tg Th17 cell adoptive transfer developed 3- to 4-fold higher levels of IFN-γ producing *T*. *cruzi*-specific T cells detectable responses upon stimulation with either p7 or whole TS antigen. (C-D) Adoptive transfer of TS-CD4-Tg Th1 cells resulted in an approximately 3-fold increase in antibody titers, and adoptive transfer of Th17 cells resulted in an approximately 9-fold increase, as measured by endpoint titers.

In order to test whether Th17 cells can have similar *in vivo* helper effects for CD8^+^ T cells in a physiological setting, where millions of CD4^+^ and CD8^+^ T cells of diverse specificities co-exist, we adoptively transferred immunocompetent WT BALB/c mice with TS-CD4-Tg Th1 or Th17 cells, then challenged these mice with highly virulent blood-form trypomastigotes. Total splenic cells and purified CD8^+^ T cells recovered from mice receiving Th17 cells contained 3- to 4-fold higher frequencies of antigen-specific total spleen cells (Fig 7A) and CD8^+^ T cells (Fig 7B) compared with mice given Th1 cells, consistent with our findings in RAG KO mice. These latter results indicate that superior Th17 helper effects were reproducible in a physiologic setting. In addition, WT BALB/c mice receiving adoptive transfer of either Th1 or Th17 cells developed higher titers of TS-specific antibodies than mice not receiving any cell transfer, indicating that, in addition to their robust helper effects on critically protective CD8^+^ T cells, Th17 cells also provide substantial help to *T*. *cruzi*-specific antibody-secreting B cells (Fig 7C and 7D).

### *T*. *cruzi*-reactive Th17 cells can be induced *in vivo* by vaccination

Despite being highly protective, Th17 cells are not the predominant natural CD4^+^ T cell response to *T*. *cruzi* infection. However, Th17 cells could be targeted for induction by vaccination. In the past, we have successfully induced *T*. *cruzi-*specific Th1 cells *in vivo* by vaccinating wild-type mice with recombinant TS protein (rTS) and the TLR-9 agonist CpG as an adjuvant [19]. To induce Th17 cells, we vaccinated TS-CD4-Tg mice intramuscularly with rTS combined with the TLR-1/2 agonist and Th17-skewing adjuvant Pam3CSK4 instead [32], and compared these to mice receiving rTS with CpG. *T*. *cruzi*-reactive Th1 responses could be detected by IFN-γ ELISPOT assay among splenic CD4^+^ T cells recovered from TS-CD4-Tg mice vaccinated with rTS and CpG, but not from mice vaccinated with rTS and Pam3CSK4 (S8A Fig). On the contrary, the use of Pam3CSK4 as an adjuvant induced the generation of *T*. *cruzi*-reactive Th17 cells detectable by IL-17A ELISPOT, while the use of CpG as an adjuvant did not (S8B Fig). These data indicate that endogenous, *T*. *cruzi-*specific Th17 cells can be induced *in vivo* by vaccination when the appropriate biasing adjuvants for promoting Th17 cell responses are used.

## Discussion

Recent studies demonstrating that IL-17 confers resistance against *T*. *cruzi* infection raised the possibility that Th17 cells could play a protective role, although Th1 cells are typically considered the most important CD4^+^ T cell subset for immunity against *T*. *cruzi* as well as other intracellular organisms. Th17 cells are known to be important for protection against a number of extracellular bacteria and fungi [13,33,34], but are not believed to function significantly against intracellular pathogens. In addition to the protective roles of Th17 cells in the human immune system, Th17 cells are also known to drive pathology in many autoimmune and inflammatory diseases, including multiple sclerosis and rheumatoid arthritis [8]. Previous studies demonstrating a protective function for IL-17 utilized models of global IL-17 deficiency [5,6], precluding any specific interpretation of the role of Th17 cells, as IL-17 is produced by multiple innate and adaptive cellular sources. Therefore, it remained unclear whether Th17 cells were protective or pathological during *T*. *cruzi* infection.

Our lab recently generated a line of T cell receptor transgenic mice with CD4^+^ T cells specific for a single immunodominant epitope of the *trans*-sialidase virulence factor; immune responses directed against this immunodominant antigen can be highly protective. This critical reagent has greatly advanced our ability to fully explore the roles of various CD4^+^ T cell subsets in *T*. *cruzi* infection. We studied parasite-specific Th1 and Th17 cells *in vivo* in adoptive transfer experiments and *in vitro* using macrophage infection assays and CD8^+^ T cell activation assays. With these studies, we have created strong evidence that Th17 cells can orchestrate a powerful protective response to *T*. *cruzi* infection: 100% of RAG KO mice co-transferred with Th17 cells and CD8^+^ T cells survives a normally lethal *T*. *cruzi* challenge.

Mechanistically, we discovered that through the secretion of IL-17A, Th17 cells trigger the function of NADPH oxidase to provide direct protection to infected cells. A previous report suggests that IL-17A may function to retain *T*. *cruzi* within the endosomes of host cells [35]. Our discovery that IL-17A acts through NADPH oxidase, involved in the phagocyte respiratory burst, indicates that this cytokine may direct synergistic mechanisms to promote immunity in infected macrophages. NADPH oxidase functions in the cell to produce superoxide and other ROS; these reactive products could protect against *T*. *cruzi* by directly killing intracellular pathogens or by functioning as signaling molecules to activate other protective pathways. Future investigations should focus on elucidating the detailed mechanisms of protection and the signaling events involved in IL-17 receptor triggering of NADPH oxidase activity.

In addition, we demonstrated that the major mechanism of protection provided by Th17 cells *in vivo* is the help provided to critical CD8^+^ T cells, the major effector cells responsible for *T*. *cruzi* immunity, by the secretion of IL-21. IL-21 is known to have several effects on CD8^+^ T cell function, including the enhancement and maintenance of antigen-specific proliferation and function, particularly during chronic infection [30,31,36,37]. Therefore, Th17-primed (and thus IL-21-primed) CD8^+^ T cells may offer several advantages over Th1-primed CD8^+^ T cells in the course of *T*. *cruzi* infection, making Th17 cells a highly relevant CD4^+^ T cell subset to target during the development of vaccines for Chagas disease, and perhaps for other pathogens. Additional experiments will investigate whether Th17 help for CD8^+^ T cell activation through IL-21 can generate more antigen-specific cells with more broadly reactive epitope specificity than Th1 priming.

Interestingly, in our model, neutralization of IL-17 had no detrimental effects for mice receiving Th17 cell transfer, although previous studies found that IL-17-deficient animals were more susceptible to *T*. *cruzi* infection [5–6]. Our findings that IL-17 alone can protect infected macrophages *in vitro* are consistent with these previous reports that IL-17 has protective functions. However, *in vivo*, CD8^+^ T cells are the major effector cells protective against *T*. *cruzi* infection, and through the secretion of IL-21, Th17 cells efficiently promote a robust CD8^+^ T cell response independent of IL-17. In the context of this strong CD8^+^ T cell activation, the directly protective effects of IL-17 may be secondary or dispensable. In our adoptive transfer experiments, *in vitro*-differentiated Th17 cells representing an adaptive immune response are given to mice prior to *T*. *cruzi* infection, to provide a convenient model for studies of a vaccine-induced protective state. Importantly, we have demonstrated that Th17 cells can indeed be induced by vaccination in TS-CD4-Tg mice using Th17-skewing adjuvants.

Taken altogether, these data advance our knowledge of the protective CD4^+^ T cell responses to *T*. *cruzi* infection, and illuminate the relevant T cell subsets and cytokine profiles to target in vaccination strategies. This novel understanding advances the goal of developing of a human Chagas vaccine, which could prevent 12,000 deaths per year and even more instances of disability. More broadly, these studies provide the first clear and strong evidence that Th17 cells can function significantly in intracellular as well as extracellular immunity, suggesting that Th17 cells may be an advantageous immune response to similar pathogens, and indicating the need for a revised Th1/Th2/Th17 framework. Given their newly identified role in intracellular immunity and their potent effects on CD8^+^ T cells, Th17 cells may prove to have a more diverse influence on the host immune response than previously thought. With increased understanding of their many roles and the mechanisms underlying their desired protective effects, Th17 cells could become an attractive target for host-directed therapies against various infectious pathogens.

## Materials and Methods

### Ethics statement

All animal studies were conducted in accordance with the “Guide for the Care and Use of Laboratory Animals” manual published by the National Research Council and endorsed by the Assessment and Accreditation of Laboratory Animal Care (AAALAC), with the approval of the Institutional Animal Care and Use Committee (IACUC)/Animal Care Committee (ACC), under protocol #1106, assigned by Saint Louis University’s IACUC. All mice were maintained in an AAALAC accredited facility at Saint Louis University.

### Mice

BALB/c mice (NCI Charles River Laboratories, Frederick, MD), RAG KO mice (The Jackson Laboratory, Bar Harbor, ME), and *T*. *cruzi* trans-sialidase amino acids 57–74 (TSaa57-74)-specific TCR transgenic mice on the Thy1.1 BALB/c background (TS-CD4-Tg, generated in the Hoft laboratory) were used throughout this study. *Tbx21-/-* mice (The Jackson Laboratory, Bar Harbor, ME) and IL-21R KO BALB/c mice (provided by Dr. Manfred Kopf, ETH Zurich) were used to generate CD4^+^ and CD8^+^ T cells for adoptive transfer experiments. C57BL/6 wild type mice (WT, NCI Charles River Laboratories, Frederick, MD) and B6.129S6-*Cybb* ^*tm1Din*^/J mice lacking the gp91^phox^ catalytic subunit of NADPH oxidase (gp91^phox^ KO, The Jackson Laboratory, Bar Harbor, ME) were used to generate bone marrow-derived macrophages. Mice were housed under specific pathogen free conditions. Sample sizes were based on previous experience, and sample size calculations were approved by the Saint Louis University Animal Care Committee and follow AAALAC guidelines and recommendations. All studies included age- and gender-matched groups. Although no formal blinding was done, all key experimental results were reproducible in multiple experiments and when conducted by different individuals in the laboratory.

### Parasites and challenges

The Tulahuèn strain of *T*. *cruzi* was passaged through BALB/c mice and *Dipetalogaster maximus* insects. Blood form trypomastigotes (BFT) were collected from infected mice and used for systemic challenges in BALB/c mice. Culture-derived metacyclic tryptomastigotes (CMT) were generated as described [38] and used for *in vitro* studies and for the systemic challenges of RAG KO BALB/c mice. Systemic challenges were performed by subcutaneous (s.c.) injection of 5,000 BFTs or CMTs re-suspended in in 100 μl phosphate buffered saline (PBS). *In vitro* infections of macrophages were performed by culturing cells with CMT at 5–10 multiplicities of infection (MOI).

### Murine vaccinations

We used a DNA vaccine encoding the consensus TS enzymatic domain as described previously [19], originally provided by Dr. Maurício M. Rodrigues (São Paulo, Brazil). BALB/c mice were immunized with 100 μg TS-DNA or control DNA intramuscularly (i.m.). We also vaccinated mice intranasally (i.n.) with 50 μg recombinant *trans*-sialidase plus 10 μg CpG-containing oligodeoxynucleotides (ODN) 1826 (Invivogen, San Diego, CA). For dendritic cell (DC) vaccines, splenic DC were isolated after intraperitoneal (i.p.) injection of BALB/c mice with 5x10^6^ B16 cells expressing Flt-3 ligand as previously described [39]. DC were induced to mature *in vivo* with 2 μg lipopolysaccharide given intravenously (i.v.) and harvested from spleens 16 hours later using α-CD11c microbeads (Miltenyi Biotec, Inc., Auburn, CA). The purity and activation status of DC were determined by staining for CD11c, CD86 and MHC class II, and >90% pure CD11c^+^ DC were obtained. DC were pulsed with 100 μg/ml TS peptides p7 (TSaa57-74, aa sequence KVTERWHSFRLPALVNV) and/or 100 μg/ml TSKd1 (TSaa359-367, aa sequence IYNVGQVSI) for 90 minutes at 37°C, washed and then injected i.v. into BALB/c mice (1x10^6^ cells/mouse). To induce Th1 and Th17 cells *in vivo*, TS-CD4-Tg mice were anesthetized with Ketamine/Xylazine, and 50 μg of rTS protein mixed with either 50 μg CpG or 50 μg Pam3CysSerLys4 (Invivogen, San Diego, CA) re-suspended in 100 μl volumes of PBS were injected into the left and right tibialis anterior muscles (50 μl per side).

### Production of TS-specific CD4^+^ T cell clones

We screened various 18mer peptide sequences from the consensus catalytic region of TS using IFN-ɣ ELIPSOT assays and identified an immunodominant I-A^d^-restricted CD4^+^ T cell epitope (p7, TSaa57-74, aa sequence KVTERWHSFRLPALVNV, S1 Fig, S1 Table). We prepared T cell clones as described previously [2]. Briefly, we immunized mice with TS DNA 3 times, each 2 weeks apart. Two weeks after the third immunization, spleen cells were harvested and CD4^+^ T cells purified with α-CD4 microbeads (Miltenyi Biotec, Auburn, CA). T cells were stimulated *in vitro* with recombinant TS protein or irradiated spleen cells pulsed with TSaa57-74 (p7) in the presence of 10 U/ml IL-2. After 2 cycles of antigen stimulation and IL-2 expansion, T cell cloning was performed by limiting dilution.

### Generation of TS-CD4-Tg mice

We amplified clonal TCR-α and TCR-β chain cDNA from a TS p7-reactive T cell clone. We then subcloned these TCR-α (Vα2) and TCR-β (Vβ8.2) sequences into a CD2 vector (VAhCD2 vector, kindly provided by Dr. Richard DiPaolo and Dr. D. Kioussis) [40]. We digested and gel purified the TCR DNA fragments and injected them into the pronucleus of C57BL/6 x BALB/c fertilized eggs, with the help of Dr. Mike White at Washington University, Saint Louis, MO. We screened weanlings by PCR for the TCR chains Vα2 and Vβ8.2. We backcrossed TCR Tg mice onto a congenic Thy1.1^+^ BALB/c background to generate TCR transgenic peripheral T cells capable of recognizing the I-A^d^-restricted p7 epitope. Subsequent generations of TCR Tg mice were screened by flow cytometry for Vβ8.2 surface expression on peripheral T cells or MHC II:KVTERWHSFRLPALVNV tetramer staining (NIH Tetramer Core Facility, Emory University, Atlanta, GA).

### Generation of Th1 and Th17 cells

*Trans*-sialidase-specific T cell lines were derived from naïve TS-CD4-Tg mice. Spleens from naïve Thy1.1^+^ transgenic mice were harvested, and CD4^+^ T cells were purified by positive selection using α-CD4 microbeads (Miltenyi Biotec, Auburn, CA). The CD4^+^ T cells were stimulated with either α-CD3/α-CD28-coated plates or with irradiated CD4^+^ and CD8^+^ depleted Thy1.2^+^ BALB/c splenocytes pulsed with TSaa57-74 (p7) at 2.5 μg/ml. Th1 biasing conditions included 10 ng/ml recombinant mouse IL-12 (Genetics Institute, Cambridge, MA) and 10 μg/ml anti-IL-4 neutralizing mAb 11B11 (NCI Biological Resources Branch, Frederick, MD). Th17 biasing conditions included 1 ng/ml recombinant human TGF-β (Biolegend, San Diego, CA), 50 ng/ml recombinant mouse IL-6 (Invitrogen, Carlsbad, CA), 20 ng/ml recombinant mouse IL-23 (R&D Systems, Minneapolis, MN), 10 μg/ml anti-IL-4 (NCI Biological Resources Branch), and 10 μg/ml anti-IFN-γ neutralizing mAb XMG1.2 (BD Pharmingen, San Diego, CA). Every 3 days, Th1 cell cultures received 10 U/ml IL-2 while Th17 cell cultures received 20 ng/ml IL-23. Cells were stimulated again 1 week later in the presence of the same biasing cytokines and antibodies. IL-2 or IL-23 was again added 3 days after the second stimulation. Th1 and Th17 cells were used one week after the second stimulation. Polyclonal Th17 cells were generated similarly using wild-type CD4^+^ T cells; on days 0 and 7, these cells were stimulated with immature DCs pulsed with the supernatants of repeatedly freeze-thawed *T*. *cruzi* trypomastigotes. IL-17^+^ cell purification was performed using Miltenyi IL-17 cell secretion purification kits according to manufacturer instructions.

### Adoptive transfers and assessment of protective immunity

*In vitro* generated Th1 or Th17 cells were transferred i.v. into RAG KO mice, along with polyclonal CD8^+^ T cells purified from naïve BALB/c mice by positive selection using α-CD8 microbeads (Miltenyi Biotec, Auburn, CA). RAG KO mice received 0.5–2 x 10^6^ Th1 or Th17 cells, and 3–5 x 10^6^ CD8 T cells, by tail vein injection and were challenged s.c. one day later with 5,000 CMT. Parasitemia was measured post-infection microscopically using 1.5 μl peripheral blood taken from the tip of the tail. Protection was also assessed by survival of infected mice. At 3, 6, 7 and 10 days post-infection, the spleens from representative mice were harvested for immune studies.

### *In vivo* IL-17A neutralization

CD8^+^ T cells were transferred along with TS-CD4-Tg Th17 cells into RAG KO mice that were infected s.c. the following day with 5,000 *T*. *cruzi* CMT. In order to neutralize IL-17A, mice were injected intraperitoneally (i.p.) with 100 μg of either anti-mouse IL-17A mAb (clone 17F3) or mouse IgG1 isotype control (clone MOPC-21), both from Bio X Cell (West Lebanon, NH). Mice were injected with antibodies every 48 hours, beginning 1 day before infection and continuing for 30 days post infection.

### IL-17A adenovirus

A recombinant adenovirus expressing murine IL-17A was originally described by Schwarzenberger et al. [41] and generously provided by Dr. Shabaana Khader (Washington University, Saint Louis, MO). A control recombinant adenovirus (Ad5) expressing β-galactosidase (β-gal) was also used [42,43]. Viruses were propagated in HEK-293A cells at Saint Louis University using endotoxin-free conditions, purified by CsCl as previously described [44], and stored at -80°C in 20mM Tris (pH 8) with 4% sucrose. RAG KO mice were injected i.v. with 5x10^9^ PFU of recombinant adenovirus encoding either mIL-17A (IL-17A AdV) or β-gal (ctrl AdV) one day prior to infection and 7 days post-infection. Mice also received CD8^+^ T cells i.v. and were challenged s.c. with 5,000 *T*. *cruzi* CMT. IL-17A was measured in the serum of infected mice by ELISA according to the manufacturer’s instructions (BioLegend, San Diego, CA).

### Flow cytometry and intracellular cytokine staining

Cell surface staining was performed according to standard procedures using antibodies against murine CD3, CD8, CD4, CD19, CD44 and Thy1.1 (CD90.1), all purchased from BD Pharmingen (San Diego, CA). For intracellular staining, monoclonal antibodies against IFN-γ (BD Pharmingen), IL-17A, IL-17F, MIP-1α, RORγt and T-bet (eBioscience, San Diego, CA) were used. For intracellular staining of *in vitro* cultures, cells were restimulated with phorbol-12-myristate 13-acetate (PMA, 10 ng/ml) and ionomycin (500 ng/ml) for 3 hours. For intracellular staining of spleen cells post-infection, cells were restimulated *ex vivo* with A20-TS for 6 hours. All intracellular stained cells were cultured with monensin (GolgiPlug, 1 μl/ml) and brefeldin A (GolgiStop, 0.67 μl/ml) for the last 3 hours of stimulation at 37°C (both from BD Pharmingen). After surface staining, cells were fixed and permeabilized with Foxp3/Transcription Factor Staining Buffer Set (eBioscience) before intracellular staining, according to manufacturer’s instructions. Cells were analyzed with a BD LSR II flow cytometer and FlowJo v7 software (Tree Star, Inc.).

### *In vitro* protection assays

To generate peritoneal exudate macrophages (PEM), BALB/c mice were injected i.p. with 100 μg Concanavalin A (Sigma Aldrich, Saint Louis, MO). Four days later, PEM were harvested from the peritoneal cavity of these mice and plated in 8-well tissue culture slide chambers (Nunc LabTek from Thermo Scientific, Waltham, MA) at 1.25x10^6^ cells/well in DMEM with 10% FBS. After 2–5 hours, nonadherent cells were washed away and PEM were infected with *T*. *cruzi* CMT at an MOI of 5 (6.25x10^6^ parasites/well) for 3 hours. Extracellular parasites were then washed away and TS-CD4-Tg Th1 or Th17 cells added at a macrophage to T cell ratio of 25:1 (5x10^4^ cells/well). Two days later, slide chambers were removed from slides, and slides were washed, dried, fixed and stained with a modified Wright Giemsa stain, Diff-Quik (IMEB, Inc., San Marcos, CA). The number of infected macrophages was determined microscopically. Variations of this assay included using neutralizing antibodies against IFN-γ (clone XMG1.2, BD Pharmingen) or IL-17A (clone TC11-18H10, BD Pharmingen) at 10 μg/ml, using the purified cytokines mIFN-γ (1000 U/ml, Genentech, San Francisco, CA) or recombinant mouse IL-17A (100 ng/ml, R&D Systems) instead of T cells, and inhibiting inducible nitric oxide synthase (iNOS) with N6-(1-Iminoethyl)-L-lysine (L-NIL, 1mM, Sigma Aldrich, Saint Louis, MO). Supernatants from the infected macrophage cultures were collected 24 and 48 hours post infection. Nitric oxide concentration was estimated by measuring nitrite concentration in supernatants with the Griess reagent system according to the manufacturer’s instructions (Promega, Fitchburg, WI).

For experiments with bone marrow-derived macrophages (BMDM), bone marrow was harvested from the femurs and tibiae from C57BL/6 WT OR C57BL/6 gp91^phox^ KO mice. Cells were cultured on 96-well and 6-well plates with 20 ng/ml recombinant mouse M-CSF (PeproTech, Rocky Hill, NJ) in DMEM with 10% FBS for 7 days. BMDM were then removed from culture plates with CellStripper non-enzymatic dissociation reagent (Corning, Corning, NY), transferred to 8-well slide chambers at 2x10^5^ cells/well, and infected with CMT at MOI of 10. Three hours post infection, extracellular parasites were washed away and either IFN-γ or IL-17A was added. Two days later, slides were washed, dried, fixed and stained as described above.

### CD8^+^ T cell *in vitro* activation assay

Polyclonal CD8^+^ T cells were purified from naïve BALB/c mice by positive selection with α-CD8 microbeads (Miltenyi Biotec), labeled with the cell-staining dye CFSE (Invitrogen) and stimulated with a suboptimal dose of plate-bound α-CD3 antibody (1 μg/ml, clone 145-2C11, BD Biosciences) in 96-well plates with 2x10^5^ cells/well. Further activation/co-stimulation was provided by DCs (purified from spleens with α-CD11c microbeads, Miltenyi Biotec) and/or TS-CD4-Tg Th1/Th17 cells (1x10^5^ cells/well). Cellular responses were analyzed 5 days post-activation by re-stimulating with PMA and ionomycin and analyzing by flow cytometry to measure proliferation (by CFSE dilution), CD44 surface expression, and production of IFN-γ and MIP-1α. Additional modifications of this assay included using supernatants from activated Th1 or Th17 cells with or without a neutralizing antibody directed against IL-17A at 10 μg/ml (BD Pharmingen). CD8^+^ T cells were also activated with supernatants by purified recombinant mouse IL-17A, IL-17F, IL-21, IL-22 or GM-CSF at 100 ng/ml (all from R&D) instead of by co-culture with CD4^+^ T cells. Results are presented as either frequency of CD8^+^ T cells positive for the indicated activation marker or as fold increase as compared to that with α-CD3 activation alone. IL-21 concentration in supernatants was measured by ELISA according to manufacturer’s instructions (eBioscience).

### Enzyme-linked immunospot (ELISPOT) assay

Millititer HA 96-well microtiter plates with nitrocellulose bases (Millipore, Bedford, MA) were coated with 10 μg/ml of either murine anti-IFN-γ mAb (clone R46A2, BD Pharmingen, San Diego, CA) or anti-IL-17A mAb (clone TC11-18H10, BD Pharmingen) overnight at 4°C. Plates were washed with PBS 4 times and blocked with RPMI + 10% FBS at room temperature (RT) for at least 2 hours. Total spleen cells from infected RAG KO mice, spleen cells from immunized BALB/c mice, CD4^+^ T cell clones or peripheral blood mononuclear cells (PBMCs) from TS-CD4-Tg mice (1-3x10^5^ cells/well) were stimulated with negative control A20 cells (NC A20), stably TS-transfected A20 cells (TS A20), A20 cells pulsed with TS p7 (KVTERWHSFRLPALVNV at 2.5 μg/ml; A20 + p7) or A20 cells pulsed with other overlapping peptides (at 2 μM). Some ELISPOT assays included stimulations of Tg cells with peritoneal exudate macrophages infected with *T*. *cruzi*. Additional stimulations were done with L-cells transfected with I-A^d^ or I-E^d^ molecules (courtesy of Dr. Ron Germain, NIH). Numbers of IFN-γ- or IL-17A-producing cells were detected with biotinylated anti-IFN-γ Ab (clone XMG, BD Pharmingen) or anti-IL-17A (clone TC11-8H4, BD Biosciences), streptavidin conjugated to horseradish peroxidase (HRP) (Jackson Immunoresearch Laboratories, West Grove, PA) and 3-amino-9-ethylcarbazole substrate precipitation. Results are reported as number of spot-forming cells (SFCs).

### *T*. *cruzi* TS-specific IgG serum ELISA

Nunc Maxisorp flat-bottom 96-well plates (eBioscience) were coated with 5 μg/ml of recombinant *trans*-sialidase protein at 4°C overnight. Plates were washed with PBS-T and blocked with 10% FBS at room temperature, then mouse serum samples serially diluted in PBS were added to wells and incubated overnight. Plates were washed and incubated with an anti-mouse IgG antibody conjugated to horse radish peroxidase to detect TS-specific antibodies. Plates were developed with TMB substrate, and the reaction was stopped with H_2_SO_4._ Plates were analyzed at 450 nm with a reference of 540 nm, and endpoint titers were determined as OD readings above the highest OD measured for uninfected controls.

### Statistics

Statistical analyses were performed using Prism v4 software (GraphPad Software, Inc., La Jolla, CA). Mann-Whitney U tests or unpaired Student t tests were used to compare responses between groups. Log-rank (Mantel-Cox) and Gehan-Breslow-Wilcoxon tests were used to compare survival between groups. All data are presented with standard errors, ranges or scatter plots to allow easy assessment of the variation.

## Supporting Information

S1 Fig Identification of an immunodominant *trans*-sialidase MHC-II-restricted T cell epitope. Previous reports indicated the presence of CD4 epitopes within the N-terminal region of the TS catalytic domain (aa33-275) [45]. (A-C). We designed and synthesized 57 overlapping 18mers spanning this region with 14 amino acid overlaps (see S1 Table). Spleen cells from control (A), DNA-TS intramuscularly (i.m.) (B) or CpG-rTS intranasally (i.n.) (C) vaccinated mice were mixed with APCs pulsed with individual peptides and added to IFN-γ ELISPOT wells. Peptide #7 (TSaa57-74, KVTERVVHSFRLPALVNV) consistently induced IFN-γ production in T cells from TS DNA and CpG-rTS vaccinated mice (B-C). (D) To identify the restriction element of p7, we prepared TS-specific CD4^+^ T cell lines from DNA-TS vaccinated mice. L cells transfected with various MHC elements (I-A^d^, I-E^d^, or AβEα^d^) were pulsed with p7 and mixed with TS-specific CD4^+^ T cells in IFN-γ ELISPOT assays. p7 is restricted by I-A^d^. (E) APCs pulsed with different doses of p7 (0.01–10μg/ml) demonstrate high avidity of peptide binding to MHC. (F) Presentation of this peptide during *T*. *cruzi* infection is shown, where TS-specific CD4^+^ T cells were stimulated with macrophages (PEMs) pulsed with p7 or infected with *T*. *cruzi* parasites in overnight IFN-γ ELISPOT assays.
(TIF)

S2 Fig TCR Tg CD4^+^ T cells specific for p7, a newly discovered *Trypanosoma cruzi trans*-sialidase epitope, help induce protective immunity. (A) Schematic model of TS and positions of immunogenic CD4 p7 and CD8 TSKd1 epitopes. (B) Survival results of BALB/c mice (>5/group) challenged with *T*. *cruzi* trypomastigotes after vaccination with DC pulsed with the CD4 p7 and/or the CD8 TSKd1 peptide. Vaccination with both peptides was significantly more protective than vaccination with either peptide alone (p<0.05 by both two-tailed Fisher exact test and Kaplan-Meier analysis). (C) Schematic of *T*. *cruzi* p7-specific TCR transgenic (TS-CD4-Tg) mice generation. (D) T cells from TS-CD4-Tg mice responded in IFN-γ ELISPOT assays to APCs (A20 cells) either transfected with TS (TS A20) or pulsed with p7 peptide (A20 + p7). (E-F) CFSE-labeled T. cruzi p7-specific TCR Tg CD4^+^ T cells were transferred i.v. into naïve congenic BALB/c mice and activated in vivo by TS DNA intramusculary or 5,000 *T*. *cruzi* trypomastigotes subcutaneously twice one week apart. Representative Flow Cytometry plots depicting the frequency of Thy1.1^+^ CD4^+^ Tg T cells (E) and the absolute number of proliferating Tg spleen cells (F) 3 days after the second stimulation are shown.
(TIF)

S3 Fig Generation of *T*. *cruzi*-specific Th1 and Th17 cells. (A) A schematic of the protocol for generation of Th1 and Th17 cells is shown. Splenic CD4^+^ T cells from TS-CD4-Tg mice were purified by positive selection and stimulated with either α-CD3/α-CD28-coated plates or with irradiated splenocytes depleted of CD4^+^ and CD8^+^ cells and pulsed with p7. The T cell cultures were biased for Th1 responses with 10 ng/ml IL-12 and 10 μg/ml α-IL-4, and for Th17 responses with 1 ng/ml TGF-β, 50 ng/ml IL-6, 20 ng/ml IL-23, 10 μg/ml α -IL-4, and 10 μg/ml α-IFN-γ. Every 3 days, Th1 cell cultures received 10 U/ml IL-2 while Th17 cell cultures received 20 ng/ml IL-23. Cells were re-stimulated 1 week later in the presence of the same biasing cytokines and antibodies and used one week after the second stimulation. (B-C) ICS of Th1 and Th17 cells showing production of IFN-γ and IL-17A. (D-E) ICS of Th1 and Th17 cells showing expression of the transcription factors T-bet and RORγt. (F) IL-21 was produced by RORγt^+^, IL-17A^+^ cells among the CD4^+^ T cells cultured under Th17-skewing conditions.
(TIF)

S4 Fig Adoptively transferred Th17 cells retain IL-17-producing capabilities post-infection. Th1 or Th17 cells were co-transferred with polyclonal CD8^+^ T cells into RAG KO mice prior to *T*. *cruzi* infection. 3, 6 and 10 days post-infection, spleen cells were harvested and the CD4^+^ T cell responses were studied. (A-B) Total spleen cells were restimulated *ex vivo* with TS A20 antigen presenting cells for 6 hours and analyzed by intracellular cytokine staining for IFN-γ (A) and IL-17A (B). Shown are the frequencies of TS-specific, cytokine-producing cells as a percentage of CD4^+^ T cells. (C-D) Total spleen cells were restimulated *ex vivo* with A20 cells pulsed with p7 overnight in ELISOT assays to identify IFN-γ-producing (C) or IL-17A-producing cells (D) Shown is the number of p7-specific spot forming cells per million total spleen cells after subtracting background (SFC to negative control A20 cells). Similar results were seen in multiple experiments.
(TIF)

S5 Fig Purified IL-17^+^ Th17 cells protect against *T*. *cruzi*. (A) More than 90% of cells captured using purification kits expressed IL-17, indicating high purity. (B) The persistence of adoptively transferred Th17 cells could be detected among spleen cells recovered from mice receiving either total Th17 cell cultures or purified IL-17^+^ T cells more than one week later, as indicated by spots formed on IL-17 ELISPOT in response to p7, the cognate epitope for the TCR transgenic T cells. (C) Similar levels of IFN-γ were produced by spleen cells upon stimulation with TS between mice receiving either total Th17 cells or purified IL-17^+^ Th17 cells, indicating similar immune activation. (D). Mice receiving purified IL-17^+^ Th17 cells controlled infection as well as mice receiving total Th17 biased cells, as indicated by similar blood parasite burden. (E) Co-transfer of purified IL-17^+^ Th17 cells protected mice from mortality following *T*. *cruzi* challenge.
(TIF)

S6 Fig Polyclonal parasite-specific Th17 cells generated from wild-type CD4^+^ T cells confer significant protection against *T*. *cruzi-*infection. (A) Polyclonal parasite-specific Th17 cells can be generated by co-culturing wild-type CD4^+^ T cells with dendritic cells pulsed with whole *T*. *cruzi* parasite lysate antigens twice, one week apart under Th17-skewing conditions, as indicated by TS antigen-specific IL-17A secretion in ELISPOT assays at week 2. (B) Purified CD8^+^ T cells recovered from RAG KO mice co-adoptively transferred with polyclonal, parasite-specific Th17 cells exhibit increased expression of activation markers, including IFN-γ, T-bet, and CD44, compared to RAG KO mice not receiving co-adoptive transfer of polyclonal Th17 cells, as measured by ICS assay. (C) Co-adoptive transfer of polyclonal Th17 cells with CD8^+^ T cells results in improved control of parasitemia at day 18 compared to mice receiving CD8^+^ T cells alone or no T cell transfer. (D). As seen with transgenic, *T*. *cruzi*-specific Th17 cells, polyclonal Th17 cells also confer 100% long-term protection from *T*. *cruzi*-related mortality.
(TIF)

S7 Fig *T*. *cruzi*-specific T-bet KO Th17 cells confer protection similar to WT Th17 cells. (A) A small population of Tg CD4^+^ T cells cultured under Th17-biasing conditions expressed IFN-γ. T-bet KO Tg Th17 cells are unable to produce IFN-γ, as confirmed by ICS assays. (B) ICS analysis of cells recovered from mice up to 101 days after infection and stimulated *ex vivo* with TS A20 cells demonstrates that T-bet KO Th17 cells do not regain IFN-γ-producing capabilities *in vivo*. (C) Parasitemia measured up to 101 days post-infection indicated that protection by T-bet KO Tg Th17 cells was comparable to that of WT Th17 cells and was maintained long-term.
(TIF)

S8 Fig (A) Intramuscular vaccination of TCR Tg mice with recombinant TS protein (rTS) induces antigen-specific Th1 cells, detectable by IFN-γ ELISPOT, when the TLR-9 agonist CpG is added as an adjuvant, but not the TLR-1/2 agonist Pam3CSK4. (B) Antigen-specific Th17 cells, detectable by IL-17A ELISPOT assay, develop in mice vaccinated with rTS and Pam3CSK4, but not mice vaccinated with rTS and CpG. The induction of Th17 cells by vaccination with rTS and Pam3CSK4 is increased in T-bet KO TCR Tg mice. Shown are responses to A20 TS cells with background subtracted (SFC to negative control A20 cells).
(TIF)

S1 Table TS peptide amino acid sequences used in ELISPOT assays in S1 Fig. (TIF)
