# Supplementary figures and images for: Th17 Cells Are More Protective Than Th1 Cells Against the Intracellular Parasite Trypanosoma cruzi
Source: PLoS Pathog. 2016 Oct 3;12(10):e1005902. doi: 10.1371/journal.ppat.1005902 (PMC5047564; doi:10.1371/journal.ppat.1005902)

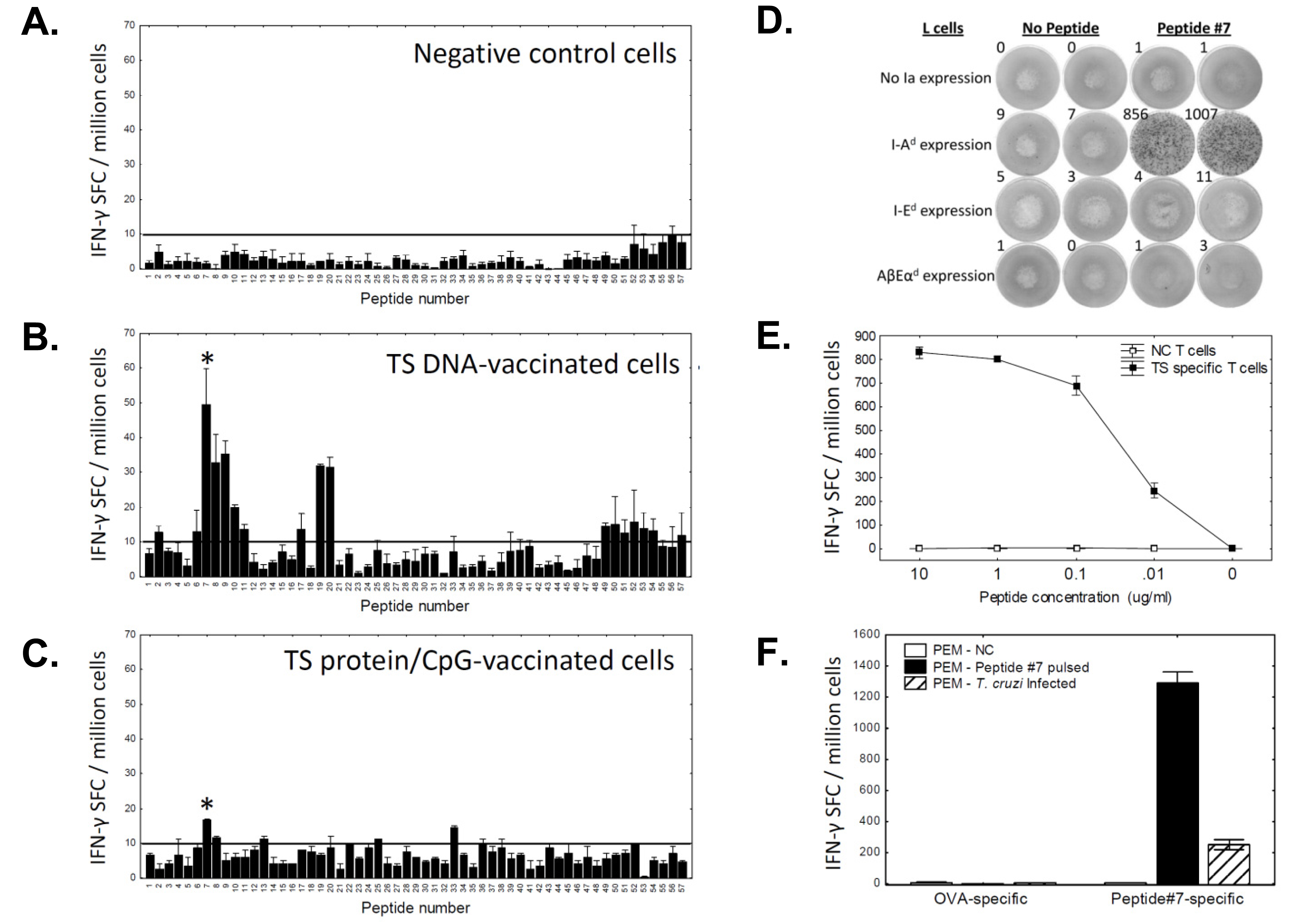

Supplement: S1 Fig — Previous reports indicated the presence of CD4 epitopes within the N-terminal region of the TS catalytic domain (aa33-275) [45]. (A-C). We designed and synthesized 57 overlapping 18mers spanning this region with 14 amino acid overlaps (see S1 Table). Spleen cells from control (A), DNA-TS intramuscularly (i.m.) (B) or CpG-rTS intranasally (i.n.) (C) vaccinated mice were mixed with APCs pulsed with individual peptides and added to IFN-γ ELISPOT wells. Peptide #7 (TSaa57-74, KVTERVVHSFRLPALVNV) consistently induced IFN-γ production in T cells from TS DNA and CpG-rTS vaccinated mice (B-C). (D) To identify the restriction element of p7, we prepared TS-specific CD4+ T cell lines from DNA-TS vaccinated mice. L cells transfected with various MHC elements (I-Ad, I-Ed, or AβEαd) were pulsed with p7 and mixed with TS-specific CD4+ T cells in IFN-γ ELISPOT assays. p7 is restricted by I-Ad. (E) APCs pulsed with different doses of p7 (0.01–10μg/ml) demonstrate high avidity of peptide binding to MHC. (F) Presentation of this peptide during T. cruzi infection is shown, where TS-specific CD4+ T cells were stimulated with macrophages (PEMs) pulsed with p7 or infected with T. cruzi parasites in overnight IFN-γ ELISPOT assays. (TIF) [file ppat.1005902.s001.tif]

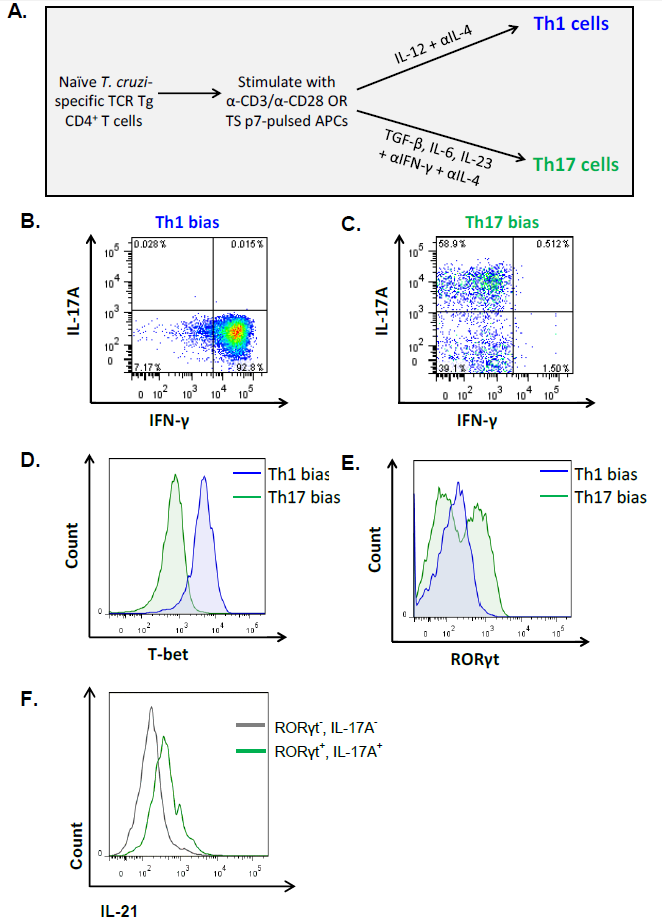

Supplement: S3 Fig — (A) A schematic of the protocol for generation of Th1 and Th17 cells is shown. Splenic CD4+ T cells from TS-CD4-Tg mice were purified by positive selection and stimulated with either α-CD3/α-CD28-coated plates or with irradiated splenocytes depleted of CD4+ and CD8+ cells and pulsed with p7. The T cell cultures were biased for Th1 responses with 10 ng/ml IL-12 and 10 μg/ml α-IL-4, and for Th17 responses with 1 ng/ml TGF-β, 50 ng/ml IL-6, 20 ng/ml IL-23, 10 μg/ml α -IL-4, and 10 μg/ml α-IFN-γ. Every 3 days, Th1 cell cultures received 10 U/ml IL-2 while Th17 cell cultures received 20 ng/ml IL-23. Cells were re-stimulated 1 week later in the presence of the same biasing cytokines and antibodies and used one week after the second stimulation. (B-C) ICS of Th1 and Th17 cells showing production of IFN-γ and IL-17A. (D-E) ICS of Th1 and Th17 cells showing expression of the transcription factors T-bet and RORγt. (F) IL-21 was produced by RORγt+, IL-17A+ cells among the CD4+ T cells cultured under Th17-skewing conditions. (TIF) [file ppat.1005902.s003.tif]

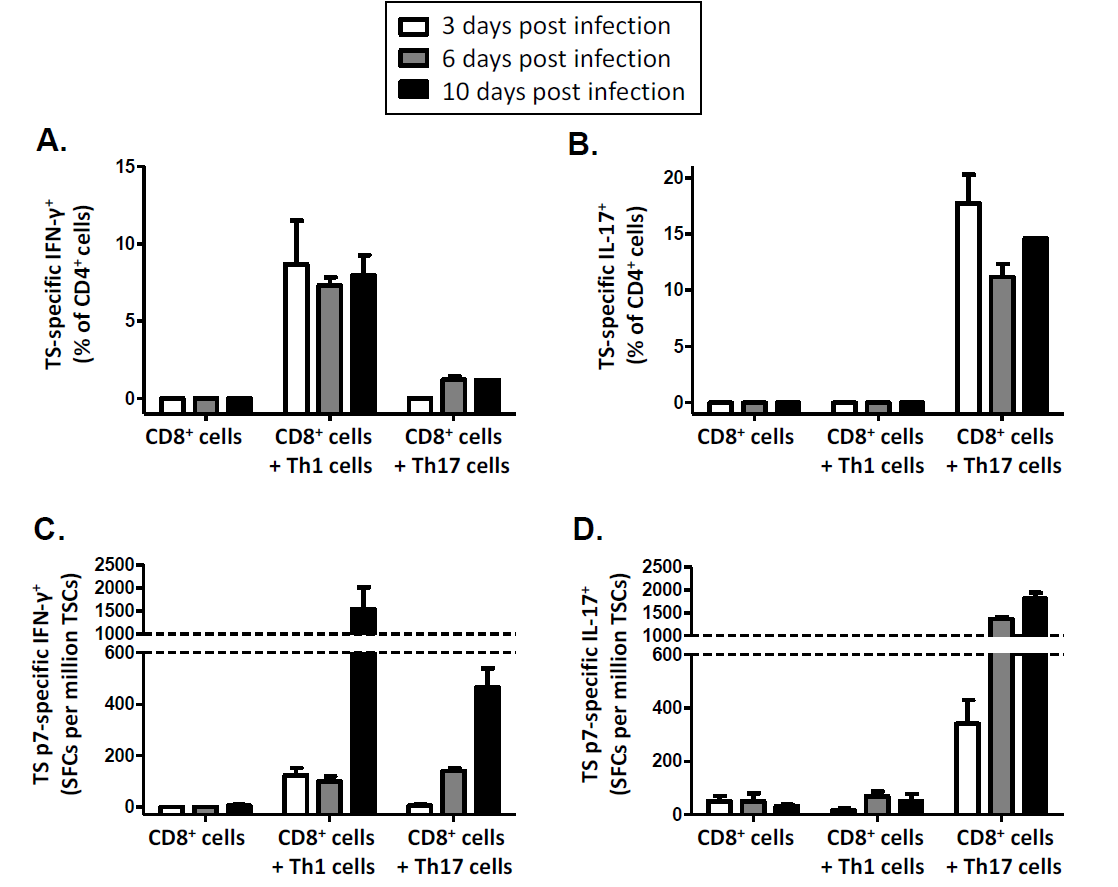

Supplement: S4 Fig — Th1 or Th17 cells were co-transferred with polyclonal CD8+ T cells into RAG KO mice prior to T. cruzi infection. 3, 6 and 10 days post-infection, spleen cells were harvested and the CD4+ T cell responses were studied. (A-B) Total spleen cells were restimulated ex vivo with TS A20 antigen presenting cells for 6 hours and analyzed by intracellular cytokine staining for IFN-γ (A) and IL-17A (B). Shown are the frequencies of TS-specific, cytokine-producing cells as a percentage of CD4+ T cells. (C-D) Total spleen cells were restimulated ex vivo with A20 cells pulsed with p7 overnight in ELISOT assays to identify IFN-γ-producing (C) or IL-17A-producing cells (D) Shown is the number of p7-specific spot forming cells per million total spleen cells after subtracting background (SFC to negative control A20 cells). Similar results were seen in multiple experiments. (TIF) [file ppat.1005902.s004.tif]

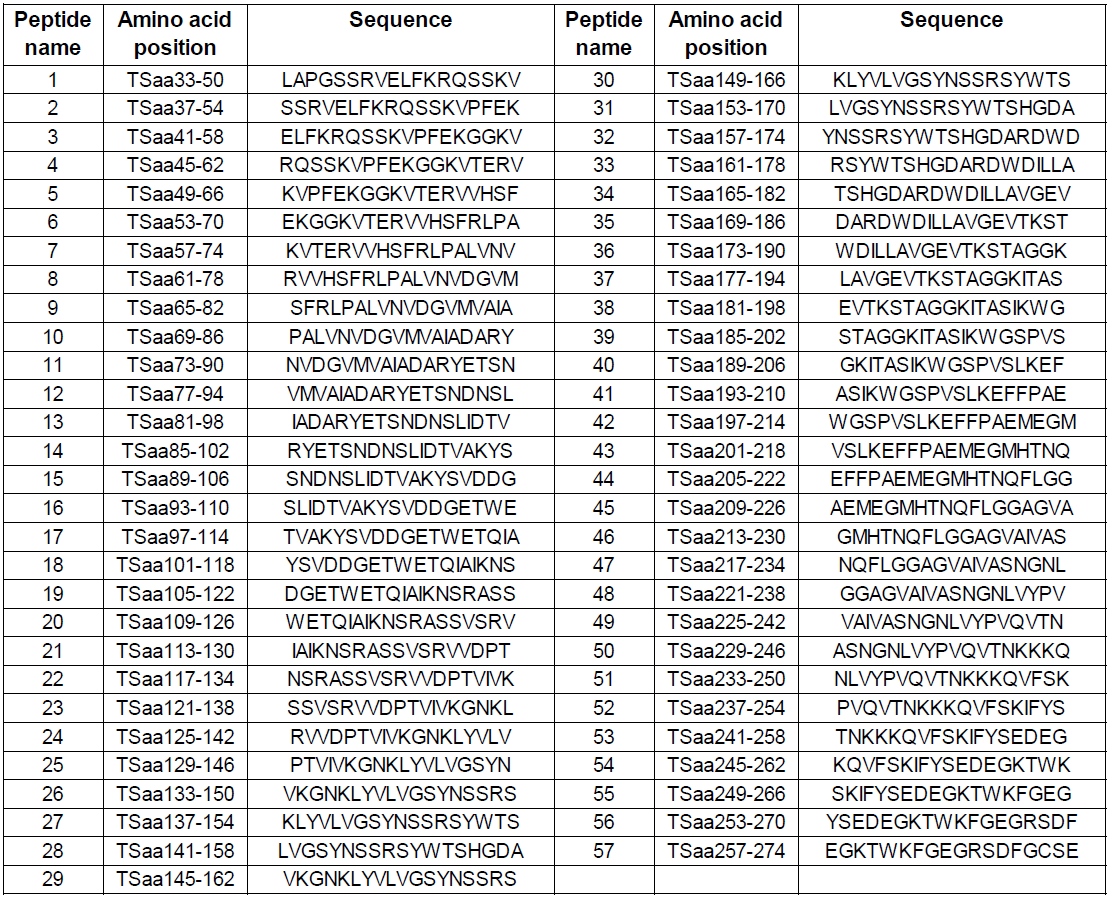

Supplement: S1 Table — (TIF) [file ppat.1005902.s009.tif]
